# Comparison of canine ECV measurements derived from CMR: bolus injection vs. slow infusion of Gd-BOPTA

**DOI:** 10.1186/1532-429X-16-S1-P64

**Published:** 2014-01-16

**Authors:** Kyungpyo Hong, Eugene G Kholmovski, Christopher J McGann, Ravi Ranjan, Daniel Kim

**Affiliations:** 1 UCAIR, Radiology, University of Utah, Salt Lake City, Utah, USA; 2 Division of Cardiology, Internal Medicine, University of Utah, Salt Lake City, Utah, USA

## Background

Myocardial extracellular volume (ECV) fraction [1], derived from pre- and post-contrast cardiac T_1 _measurements, is an emerging biomarker of diffuse cardiac fibrosis. The major benefits of ECV over post-contrast cardiac T_1 _is that the former is less sensitive to confounders such as contrast agent type and dosage, specific delayed imaging time, renal function, and magnetic field strength. Recent studies using inversion-recovery (IR) based T_1 _mapping in control human subjects[2] and patients[3] reported that ECV measured with a bolus injection of contrast agent agrees well with ECV measured with a slow infusion of contrast agent. It is unknown if this approximation holds in canines which are frequently used in preclinical research. We sought to compare canine ECV measurements derived from bolus and slow infusion protocols.

## Methods

Eighteen mongrel dogs with normal myocardium were imaged at 3T (Verio, Siemens). Cardiac T_1 _maps were acquired in 3 short-axis planes (base, mid, and apex) using the arrhythmia-insensitive-rapid (AIR) cardiac T_1 _mapping pulse sequence based on B_1_-insensitive saturation-recovery of magnetization preparation [4], with the following imaging parameters: spatial resolution = 1.4 × 1.4 × 7.0 mm, temporal resolution = 217 ms, saturation-recovery time = 600 ms. The AIR acquisition was performed with "paired" consecutive phase-encoding steps in centric k-space ordering to minimize image artifacts due to eddy currents. Cardiac T_1 _maps were acquired pre-contrast, 15 min after a bolus injection of Gd-BOPTA (MultiHance; 0.15 mmol/kg), and 30 min after slow infusion of Gd-BOPTA (0.002 mmol/kg/min). Blood samples were drawn during MRI for hematocrit calculation. For image analysis, myocardium and blood pool were manually segmented, and myocardial and blood T_1 _were calculated. Subsequently, ECV was calculated as ECV=(1-hematocrit)x(ΔR_1, myocardium_/ΔR_1, blood_), where R_1 _is T_1_^-1^, and Δ is the difference between post- and pre-contrast. Linear regression and Bland-Altman analyses were performed to compare the results, and magnitude of effect size of the observed difference was calculated using Cohen's d from t-tests.

## Results

**Figure 1:**
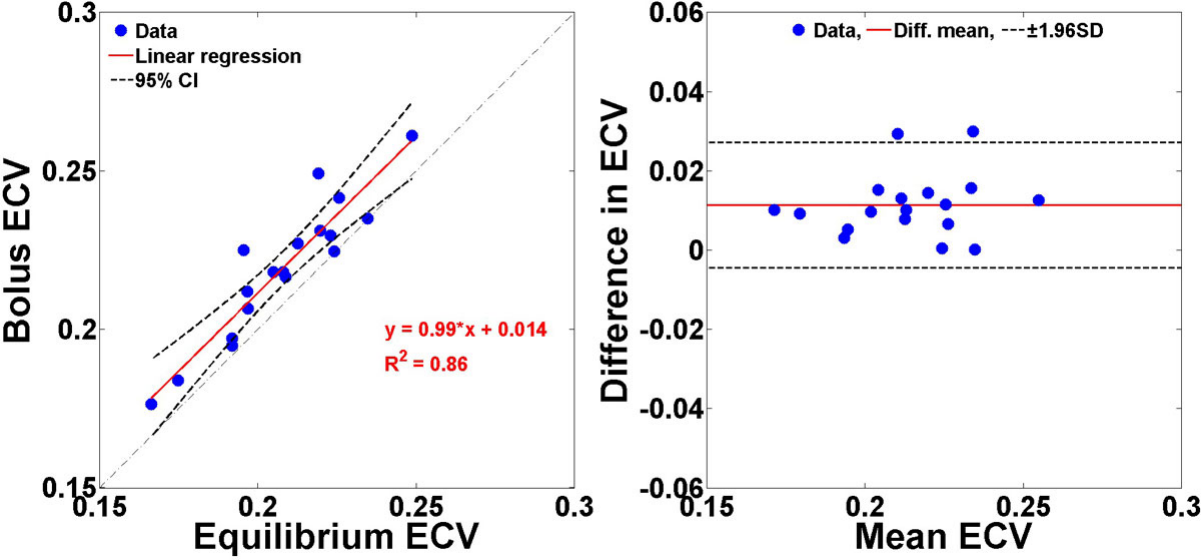
**(left) Linear regression and (right) Bland-Altman analyses comparing ECV measurements between bolus and slow infusion protocols**.

As shown in Figure 1, ECV measurements derived from the two different injection protocols were strongly correlated (Pearson's R = 0.93, p < 0.001) and in fair agreement (mean difference = 0.011, upper and lower 95% limits = 0.027 and -0.0045), which corresponds to a medium effect size in difference (d = 0.55).

## Conclusions

This study suggests that the contrast agent kinetics between canines and humans are slightly different and that in canines 15 min after a bolus injection may not be dynamic equilibrium.

## Funding

Ben B. and Iris M. Margolis Foundation.
